# Together! Clinic, research and teaching in human medicine, nursing, veterinary medicine and psychology: interdisciplinary, interprofessional, transhierarchical

**DOI:** 10.3205/zma001754

**Published:** 2025-04-15

**Authors:** Christoph Nikendei

**Affiliations:** 1 Universitätsklinik für Allgemeine Innere Medizin und Psychosomatik, Standort Bergheim, Heidelberg, Germany

## Editorial

Wow! What a wealth and what a range of great research and project work! This issue of the GMS Journal for Medical Education (GMS) immediately grabbed me – what an insight into the teaching of human medicine [1], [2], [3], [4], [5], [6], [7], [8], [9], [10], [11], nursing and healthcare [12], psychology [13] and veterinary medicine [14]. It is also pleasing that interprofessional [1], [11], interdisciplinary [2], [3], [8] and transhierarchical [5] teaching approaches are emphasised in this issue. In times of financial restrictions, a lack of specialised staff, increasing psychological strain on employees and political threats from within and without, this is a welcome sign. Interprofessional, interdisciplinary and transhierarchical work can only succeed through integration. Integration of different characters, educational biographies, countries of origin and cultures. This issue stands for this in a broader sense, and medical training must also stand for this. Especially if we want to maintain and uphold the quality of care, as reflected in the articles by Eckel et al. [4], Prediger et al. [6], Krungkraipetch et al. [7] and Tsikas et al. [10]. We can only fulfil this will to implement and these self-demands if we manage to fight against the increasing social divide [15], against the rise of right-wing populism ([https://www.deutschlandfunk.de/rechtspopulismus-rechtsextremismus-europa-rechtsruck-100.html], retrieved on 16.02.2025), against the climate crisis and for planetary health [16]. We should also pack all of this in our emergency kit, which Seiler et al. [9] from the field of general medicine provide us with in this issue. Because even if there is a serum that can save us from the “escape room”, as Lemos et al. [8] outline in their article, such a miracle serum is definitely not available to us for our society, our healthcare system and our planet. In this respect, we will have to get used to the fact that joy is always accompanied by seriousness. 

Yours sincerely,

Christoph Nikendei

## Author’s ORCID

Christoph Nikendei: [0000-0003-2839-178X]

## Competing interests

The author declares that he has no competing interests.
